# The Role of Experience and Gender in Founders' Business Planning Activities: A Meta Analysis

**DOI:** 10.3389/fpsyg.2021.689632

**Published:** 2021-08-12

**Authors:** Jiangshui Ma, Shuxing Chen, Yenchun Jim Wu, Min Shu

**Affiliations:** ^1^School of Business Administration, Faculty of Business Administration, Southwestern University of Finance and Economics, Chengdu, China; ^2^School of Economics, Southwestern University of Finance and Economics, Chengdu, China; ^3^Graduate Institute of Global Business and Strategy, National Taiwan Normal University, Taipei, Taiwan; ^4^Leisure and Recreation Administration Department, Ming Chuan University, Taipei, Taiwan

**Keywords:** business planning, managerial experience, entrepreneurial experience, gender difference, planning sophistication

## Abstract

The question of why entrepreneurs undertake business planning activities differently, ranging from planning “in the head” to generating formal written documents, is still impenetrable. Aggregating data on 11,064 observations from 32 independent data set, this study meta-analyzed how business experience and gender influence entrepreneurs' disposition to business planning behaviors. Surprisingly, contradictory to some extant views that entrepreneurs without prior experience are more likely to make business plans, we found that both managerial experience and entrepreneurial experience positively influence entrepreneurs' subsequent business planning behaviors. Drawing insight from the effectuation and institutional perspectives, this study showed that, rather than entrepreneurial experience, managerial experience motivates entrepreneurs to generate formal business plans. For entrepreneurs who create formal business plans, both entrepreneurial experience and managerial experience enhance their business planning sophistication. In addition, we examined the moderating effects of gender on the relationship between business experience and business planning. The results suggested that female entrepreneurs with entrepreneurial experience are more likely to undertake business planning behaviors and create formal business plans than their male counterparts.

## Introduction

Business planning is often taken for guaranteed as a handy tool for entrepreneurship by universities, governments, investors, and consultant agencies, as extensive studies reveal a positive association between business planning and venture performance (Delmar and Shane, 2003; Gruber, 2007; Burke et al., 2010). However, in practice, business planning is not as prevalent as we generally think, and a large portion of entrepreneurs do not undertake business planning activities or only “plan in head” (Brinckmann et al., 2010). Why do many entrepreneurs give up business planning and “just storm the castle,” while others engage in informal business planning or generate formal written business plans?

Viewed historically, the theories that have been proposed to explain entrepreneurs' disposition to business planning are inconsistent, and some arguments even propose contradictory insights. For instance, from the institutional perspective, writing a business plan is only a symbolic act taken for institutional reasons (Karlsson and Honig, 2009). Without external reasons, business plans are useless for entrepreneurs. However, from the effectuation perspective, business plans are useful instruments for entrepreneurship, but entrepreneurs are impeded from accomplishing them for the purpose of effectiveness (Sarasvathy, 2001; Dew et al., 2009). Even though whether and how to engage in business planning are considered essential issues (Baker et al., 1993; Barry, 1998), to date, the formation mechanism for business plans is still impenetrable and inconclusive.

One issue is that antecedents that influence the propensity of owner-managers to engage in business planning are poorly researched and ambiguous (Richbell et al., 2006). Tremendous extant studies focus on the plan-performance relationship rather than the business planning process. In this article, our pursuit is to theorize and empirically assess the antecedents of business planning. In particular, we focus on business experience and gender, which are perceived as critical factors. The transformation of information from prior experiences will influence entrepreneurs' perceptions and subsequent behaviors (Brinckmann et al., 2019). Furthermore, gender inequalities, such as resource restriction, risk tolerance, and work-family conflicts, also influence entrepreneurial preparedness (Yusuf and Saffu, 2005).

Furthermore, many divergent accounts have failed to explicitly differentiate business planning behaviors, lumping them all into one or two categories. This study provides an analytical framework that separates business planning behaviors into plan or not, classifying business planning activities as formal/informal planning and differentiating formal business plans by their degree of sophistication. Although a similar approach was adopted previously to investigate the plan-performance relation (Wijewardena et al., 2004; Yusuf and Saffu, 2005), to our knowledge, it has never been utilized to examine why entrepreneurs choose varied planning behaviors. Accordingly, this framework helps us comprehend the business plan formation mechanism, distinguishing the degrees of planning behaviors such as having no plan, “plan in head” or a formal written plan.

In this article, we examine whether and to what extent prior experience and gender differences impact the business planning process, providing valuable insights into entrepreneurship preparedness. Building on the theory of the institution (Karlsson and Honig, 2004) and the theory of effectuation (Sarasvathy, 2001; Dew et al., 2009), we conducted a meta-analysis on the experience–business planning relationship and specifically scrutinized entrepreneurs' distinctive prior experiences, namely, entrepreneurs' managerial experience and entrepreneurial experience. Furthermore, from the resource restriction (Bruin et al., 2006; Roomi, 2009) and risk preference perspectives, we tested gender differences in business planning by examining the moderating effect of gender.

Rather than examining the relationship between business plans and venture performance, we extend this stream of research by addressing why entrepreneurs undertake business planning and to what extent. We found that entrepreneurial experience promotes informal business planning and that managerial experience promotes formal business planning. Another contribution is that we provide an explanation complementary to the effectuation perspective by proposing that entrepreneurs assess the value and cost of business planning simultaneously. In addition, we investigated the gender difference in business planning and found that female entrepreneurs with entrepreneurial experience are more likely to undertake business planning and generate formal plans.

The next section reviews the business planning literature from the planned behavior perspective, institutional perspective, and effectuation perspective. Section Method develops the hypotheses, Section Measures provides our methodology and sample, and Section Results presents our empirical results. Finally, we conclude with a discussion and implications.

## Theoretical Background

### The Sparkling Business Plan

Entrepreneurship is well known for having a high failure rate, and it is difficult to explain why some new ventures outperform to a greater extent than others (James et al., 2005). Considering the important role of new ventures in economic growth, innovation, and job creation, scholars have made great efforts to identify the underlying mechanism for venture emergence and survival. A large amount of the existing research supports the notion that business planning positively influences new venture performance (Burke et al., 2010). Consequently, business planning is employed as a critical variable to explain why some new ventures are more successful than others in much of the extant literature. The absence or presence of a business plan in the entrepreneurship process is considered a key element of new venture survival. Entrepreneurs are generally advised to develop formal business plans (Hopp et al., 2018). Business planning is defined as the process by which entrepreneurs create a vision of the future and develop the necessary objectives, resources, and activities to achieve an entrepreneurial opportunity (Castrogiovanni, 1996; Chen et al., 2009). From the perspective of the theory of planned behavior (TPB), business plans provide unlimited benefits for new venture emergence, such as entrepreneurs' goal setting, social communication tools, entrepreneurship commitment, hazard avoidance, and legitimacy (Ajzen, 1991). Business planning serves a central capstone role in entrepreneurship and counseling (Davidsson and Gordon, 2010).

First, defining goals or making plans triggers nascent entrepreneurs' actions to create new ventures and leads to better performance (Locke and Latham, 1991; Brinckmann et al., 2010). By emphasizing learning and adaption, entrepreneurs who undertake business plans are more likely to realize gestation activities faster than others. This is because business plans restrict entrepreneurs' distraction toward irrelevant activities and increase their concentration on growth activities (Liao and Gartner, 2007). Second, through simulating the venture creation process and a dynamic environment, the business planning process, which is similar to operating a new company on paper, attenuates pell-mell development, reduces the odds of failure and limits decision-making biases (Crawford-Lucas, 1992; Delmar and Shane, 2003). By pretesting a business idea and analyzing prodigious amounts of information, entrepreneurs can update and grasp the entrepreneurial opportunity more accurately. Third, business plans, which clarify short and long-term objectives, improve new ventures' internal, and external operations by making them smoother and more transparent. Entrepreneurial activities can be clearly and precisely organized with a “minimum of distortion”(Mintzberg and Waters, 1985).

Business plans also play a critical role in venture emergence at the organizational level. On the internal side, business plans guide the entrepreneurship process, serve as a basis for subsequent strategic decisions, and boost team collaboration and employee motivation (Deakins et al., 1999). On the external side, business plans help entrepreneurs acquire legitimacy from governments and trust from partners, overcoming the liabilities of newness, and smallness (Karlsson and Honig, 2004). In addition, financiers utilize business plans as a primary tool to evaluate a business's potential and monitor a new venture's subsequent entrepreneurial activities (Kuratko and Hodgetts, 2004). Ultimately, scholars assert that business planning is the minimum requirement for entrepreneurship preparedness, which strengthens entrepreneurs' support networks, leads to obtaining credit from financial institutions, and generates positive momentum (Mazzarol et al., 2009).

Regarding the extensive benefits listed above, most academicians propose that, even if entrepreneurship success is not guaranteed, business plans contribute to venture emergence and are positively correlated with new ventures' performance (Gruber, 2007). Consequently, business planning is employed as a crucial learning instrument in entrepreneurship education and integrated into tremendous business competitions (Karlsson and Honig, 2009).

### Heterogeneous Business Planning

Although a substantial number of studies advocate the positive influence of business planning on new ventures, many entrepreneurs still do not undertake business planning behaviors (Robinson et al., 1984). The widespread use and awareness of business plans do not lead to all firms crafting one (Bhide, 2003). In practice, only some entrepreneurs engage in business planning behaviors, and a portion of them accomplish business planning through formal written documents (Wijewardena et al., 2004). In Shuman and Seeger (1986) study, 51% of the entrepreneurs did not have formal business plans when they started. Business planning is a heterogeneous activity when executed by entrepreneurs (Gruber, 2007).

Entrepreneurial ideas start with entrepreneurs' personal needs, values, beliefs, and expectations (Ramos-Rodríguez et al., 2019). They are improvisational intentions that occasionally come to peoples' minds (Greene and Hopp, 2017). Most people treat them as temporary conceptions, and they turn into nothing and are quickly forgotten. However, some individuals keep the ideas in their heads and implement basic planning activities, namely, informal business plans (Brinckmann et al., 2010). Only a portion of entrepreneurs create formally written business plans. Even though written business plans frequently contain similar sections and have similar formats, their degrees of formalization are different, and their lengths and comprehensiveness vary. Thus, business plans range from non-existent to comprehensive, formal written plans. Bracker and Pearson (1986) employed a four-level classification: unstructured plans, intuitive plans, structured operational plans, and structured strategic plans. Wijewardena et al. (2004) and Yusuf and Saffu (2005) differentiated them as no written planning, basic planning, and detailed planning.

Based on the prior research, we propose an analytical framework that categorizes business planning as having a plan or not and business planning engagement as formal (physical) or informal (mental). Furthermore, we discriminate between formal business plans (physically written) by their level of sophistication.

### Institutional Perspective

From the institutional perspective, business plans are written to comply with external pressures, such as requirements from financiers (Karlsson and Honig, 2004). According to this theory, business planning serves as a normative pressure, detracts from the action of venture creation, and has limited utility. Entrepreneurs write business plans to satisfy external demands, rather than for themselves, and business planning is a symbolic exercise and non-economically rational activity that must be endured to please stakeholders and investors (Karlsson and Honig, 2009). Once the business plans are presented, entrepreneurs loosely comply with them and have little interest in the planning documents.

### Effectuation Perspective

From the effectuation perspective, long-term goal-settings are challenging and unspecific for entrepreneurs. they prefer simple, cheap and convenient approaches which they can affect and control, rather than sophisticated plans or goals (Sarasvathy, 2001; Dew et al., 2009). Westhead and Storey (1996) argued that the absence of business planning is due to entrepreneurs being too busy surviving and having no time to plan ahead. Some “barriers” discourage or inhibit them from engaging in formal business planning behaviors, such as a lack of time and expert knowledge and a reluctance to share business ideas with others. They may think in their heads and act intuitively without a systematic business planning process (Chell, 2001).

Thus, to some extent, the effectuation theory, as an actor-based theory, is contradictory to Karlsson and Honig (2009)'s institutional theory. Entrepreneurs do not treat business planning as symbolic and irrational; instead, some barriers inhibit them from undertaking business planning and writing formal plan documents. They may intuitively make a plan in their heads or briefly write down their business ideas. Nevertheless, they may hesitate to transform their business ideas into formal written documents, which may take several months.

Although different theories are proposed to explain the distinctive behaviors related to business planning, few rigorous and in-depth empirical studies have investigated the antecedents of entrepreneurs' various planning behaviors (Brinckmann et al., 2010). Widely varying contexts are considered important antecedents for entrepreneurs' selection of behaviors, but they are difficult to examine systematically (Burke et al., 2010). In addition, it has been proposed that entrepreneurs' propensities explain their varying business planning behaviors. It has been argued that entrepreneurs' prior experiences could be a significant factor in their subsequent business planning behaviors (Brinckmann and Kim, 2015).

## Hypotheses

### Do Entrepreneurs With Prior Experience Engage in More or Less Planning?

Many scholars have discovered that individuals' disposition to engage in business planning is notably influenced by their prior knowledge and experiences (Mengel and Wouters, 2015; Brinckmann et al., 2019). Individuals' distinctive knowledge, skills, and abilities differentially affect their business planning behavior (Dencker et al., 2009). Thus, do entrepreneurs with prior business knowledge engage in more or less planning than entrepreneurs with no such knowledge? (Borges et al., 2013). For this question, empirical findings are scant and conflicting. Two conflicting views are proposed.

On the one hand, some scholars have proposed that individuals with low levels of prior knowledge and experience are more likely to engage in higher levels of business planning (Dencker et al., 2009). This is because entrepreneurs who lack experience or are unemployed may feel that they need to seek guidance more than others and business planning can compensate for their knowledge shortage (Rotger et al., 2012). Business planning is more informative and instructive for inexperienced entrepreneurs (Burke et al., 2010). In contrast, planning would be less valuable for experienced entrepreneurs as planning activities would be expected to have less of an effect for low-novelty opportunities (Mccann and Vroom, 2015). From effectuation perspective (Sarasvathy, 2001), entrepreneurs with entrepreneurial experience might avoid planning in favor of a control-oriented approach (Read and Sarasvathy, 2005; Brinckmann et al., 2019). Furthermore, entrepreneurs with abundant experience make decisions more confidently without guidance. Their motivations to make business plans are attenuated (Borges et al., 2013).

However, the faultiness of this viewpoint lies in its inconclusiveness as to whether prior business knowledge can be applied to subsequent entrepreneurship processes. In practice, entrepreneurship processes are impossible to replicate (Cope, 2005). In addition, business planning is not only implementing prior knowledge but also collecting real-time information from the business market (Shane and Delmar, 2004). Furthermore, entrepreneurship is a dynamic and multifaceted process with multiple dimensions (Gruber, 2007). Thus, even individuals who are professionals in one field still need to learn other skills (Glaister and Falshaw, 1999). For instance, entrepreneurs with management skills need to learn and prepare for industry-related knowledge. Additionally, entrepreneurs with industry knowledge have to obtain management skills through business planning. This point of view needs to be studied further, empirically.

On the other hand, many scholars have argued that entrepreneurs with prior business knowledge and experience understand the importance of business planning processes and are more likely to engage in business planning (Dencker et al., 2009). Through business operations, they are taught that business planning is necessary for entrepreneurship accompanied by high uncertainty and extraordinary risk (Brinckmann et al., 2010). In addition, entrepreneurship education and practice both strongly advocate business planning for entrepreneurs. As business plans are introduced as a systematic, prediction-oriented, approach to venture creation (Brinckmann et al., 2010), entrepreneurs with prior knowledge are more likely to engage in business planning activities.

Thus, inconsistent with previous studies to some extent, we hypothesize that entrepreneurs with business experience in different backgrounds have higher aspirations and are more willing to engage in business planning-related activities.

H1a. Entrepreneurs' entrepreneurial experience is positively associated with their engagement in business planning activities.
H1b. Entrepreneurs' managerial experience is positively associated with their engagement in business planning activities.

### Formal or Informal Business Planning

To deepen our understanding of individuals' various activities in business planning, we explored why some entrepreneurs prefer formal business plans while others choose informal business plans through prior business experiences. The transfer of information from prior experiences influences entrepreneurs' subsequent behaviors (Brinckmann et al., 2019). Formal business plans are comprehensively written and completely detailed, while informal business plans are started with a map, picture, or even several sentences but are not elaborated into formal documents (Zhang et al., 2013).

In formal written business plans, entrepreneurs seek to provide overviews of their new ventures, including the products/services, the potential market, financial projections, implementation details, leading customers, and so on (Asah et al., 2015). The average recommended length is 40 pages, and it may take 6 months to a year to complete (Arkebauer and Mcgrawhill, 1995). Similarly, the Small Business Administration (SBA) observes that the average length of a formal completed business plan is between 30 and 40 pages. Therefore, transforming business ideas into formal written documents is a heavy burden for entrepreneurs, consuming valuable time and distracting them from critical tasks. The costs of sophisticated planning may outweigh the benefits (Brinckmann et al., 2010). However, in practice, not all nascent entrepreneurs have the capability to evaluate the potential costs of business planning (Martinez et al., 2011). Entrepreneurs who already have business experience are more likely to make a comparative analysis of “plan in head” and systematic business planning.

From the effectuation perspective, individuals with greater entrepreneurial experience might avoid planning in favor of a control-oriented approach (Wiltbank et al., 2006). Without clear and evident outcomes, entrepreneurs are motivated by entrepreneurial experience to focus more on venture creation activities, such as developing products/services, than on business planning activities (Hopp, 2015). Formal and long-tern planning consumes valuable time and distracts entrepreneurs from critical tasks (Karlsson and Honig, 2004). Considering the benefits and drawbacks, entrepreneurs who face survival issues may choose to address urgent problems rather than engage in formal business planning and may believe business ideas are not necessarily transcribed into comprehensive formal documents (Carter et al., 1996). In addition, business planning may decrease a new venture's flexibility, and strict adherence to a business plan may prevent a new venture from adapting to its dynamic environment (Dencker et al., 2009). Thus, entrepreneurs with entrepreneurial experience are more likely to weigh the cost of formal written business plans and choose to make business plans by spontaneous improvisation (Baker and Leidecker, 2001). As entrepreneurial experience drives entrepreneurs to be more concerned about post-emergence survival challenges, they are more willing to choose practical actions to solve short-term operation challenges. Ultimately, entrepreneurs with entrepreneurial experience may prefer entrepreneurial action learning behaviors rather than create formal written business plans (Chen and Pan, 2019). Such plans are *ad hoc* and intuitive (Kelmar and Noy, 1990).

By comparison, managers are more likely to develop formal business plans. They are less likely to worry about planning costs because such costs are typically covered by corporate budgets. Furthermore, formal business plans are often created to respond to internal and external pressures rather than to improve performance. Founders with managerial experience have been more exposed to these isomorphic pressures (coercive, normative, and mimetic) and are more likely to develop formal business plans (Lortie and Castogiovanni, 2015). From the institutional perspective, entrepreneurs with managerial experience are more sensitive to long-term organizational goals. As well as their exposure to normative planning forces rather than survival challenges, managerial experience drives entrepreneurs to perceive value in undertaking business planning (Richbell et al., 2006). Through planning, managerial uncertainties are reduced (Armstrong, 1982; Shrader et al., 1989). Thus, given the aforementioned analyses, we hypothesize as follows:

H2a Entrepreneurs' entrepreneurship experience is negatively associated with their engagement in formal business planning activities.
H2b Entrepreneurs' managerial experience is positively associated with their engagement in formal business planning activities.

### Degree of Planning Sophistication

In addition to formal and informal business planning behaviors, entrepreneurs behave differently in terms of business planning sophistication. It is not easy to measure informal business plans, as many of them are generated mentally and are thus invisible. However, in terms of formal written business plans, we find vast differences between entrepreneurs' business planning behaviors, ranging from plans with a minimal number of pages and basic ideas to thick documents with ingenious designs and detailed descriptions. In Bracker and Pearson (1986), four distinct levels of sophistication were identified: structured strategic plans, structured operational plans, intuitive plans and unstructured plans.

Previous research has found that planning sophistication is an important contributor to firm performance (Wijewardena et al., 2004). Similarly, Kraus et al. (2008) found that the degree of formalization of business plans has a positive influence on new firms. Thus, theoretically, once entrepreneurs start to make business plans, they are supposed to develop high-quality, sophisticated business plans. However, the degree of planning sophistication varies enormously in practice. As prior knowledge and experience positively influence planning quality (Chwolka and Raith, 2012), we argue that entrepreneurs' prior experience is an important antecedent for their degree of planning sophistication.

Specifically, managerial experience and entrepreneurial experience allow entrepreneurs to engage in business planning in an effective way (Sharon and Lowell, 2001; Dencker et al., 2009; Burke et al., 2010). This is because these types of experience give entrepreneurs a better understanding of the multiple dimensions of launching and operating a new venture. Thus, given the aforementioned analyses, we hypothesize as follows:

H3b Entrepreneurs' entrepreneurship experience is positively associated with the degree of their business planning sophistication.
H3b Entrepreneurs' managerial experience is positively associated with the degree of their business planning sophistication.

### Gender Differences

Differences in entrepreneurial behaviors based on entrepreneurs' gender are apparent across countries, and entrepreneurs' business planning activities are likewise varied (Dant et al., 1996). To our knowledge, few study examined the interaction of experience and gender on business planning. Whether business experience influences male entrepreneurs and female entrepreneurs equally or not has been little discussed. However, gender interaction is adopted as an important factor to explain entrepreneurial activities in previous research. For instance, Chowdhury and Endres (2005) found education playing a more significant role for females than for males. Wilson et al. (2007) examined the interaction effect of gender and self-efficacy on entrepreneurial intentions. Accordingly, some scholars have attempted to ascertain the difference between males and females in business planning and to explore the factors that account for the variety in the activities (Yusuf and Saffu, 2005). To deepen our knowledge, we investigated the moderating effect of gender on the relationship between prior knowledge and business planning.

Female entrepreneurs are subjected to substantial gender inequality in entrepreneurship process. First, female entrepreneurs in particular are more restricted in their access to economic resources, including financial capital, information, and technology (Bruin et al., 2006; Roomi, 2009). Consequently, female entrepreneurs start their businesses with fewer resources and face higher uncertainty and risk than their male counterparts (e.g., Carter et al., 1996; Hunt and Bygrave, 2009). Such gender inequality in entrepreneurship compel female entrepreneurs to be more aware of their vulnerabilities in venture creation and more concerned about entrepreneurial preparedness. Furthermore, women have been found to be less ambitious and to have lower risk tolerance than men (Cardella et al., 2020). Similarly, (Margaça et al., 2020) found that female entrepreneurs possess a tendency toward taking less risk. Thus, entrepreneurial experience enhances female entrepreneurs' risk awareness, and they are more likely to engage in business planning activities to avoid uncertainty and decrease future risk (Brinckmann et al., 2010).

Comparatively, the managerial experience is more social and suitable for females. Female entrepreneurs tend to engage in relatively underperforming sectors that exhibit lower growth levels. Specifically, female entrepreneurs with managerial experience are less likely to take important posts, and they are less sensitive about the dynamic process of venture creation. Therefore, rather than entrepreneurial experience, which enhances female entrepreneurs' business planning activities, managerial experience has no significant differential influence on males and females.

H4a Entrepreneurial experience has a significant differential impact on male and female entrepreneurs in terms of their engagement in business planning activities. Female entrepreneurs with entrepreneurial experience will be more likely to undertake business planning activities than male entrepreneurs with entrepreneurial experience.
H4b Managerial experience has no significant differential impact on male and female entrepreneurs in terms of their engagement in business planning activities.

Female entrepreneurs with entrepreneurial experience have been found to be alarmed by resource restriction and work-life conflict (Duxbury and Higgins, 2001). For instance, as female entrepreneurs cope with family commitments, they have to make sacrifices, and their firms' employment size, revenue level, and net income are constrained (Jennings and Mcdougald, 2007). They realize that they face higher uncertainty and more risk than their male counterparts, (e.g., Carter et al., 1996; Hunt and Bygrave, 2009). Lacking resources and legitimacy, female entrepreneurs are left feeling very vulnerable and insecure. In addition, their diffidence is often mistaken as thoughtlessness. To lower their amount of risk-taking and avoid thoughtlessness, female entrepreneurs are more likely to engage in entrepreneurial preparedness and undertake systematic business planning.

Furthermore, formal business plans are often created to respond to internal and external pressures rather than to improve performance. Female entrepreneurs who have insufficient social networks may face higher pressure, because those social networks tend to be male-dominated (Aidis et al., 2008). Nevertheless, formal written documents, which can be passed around to investors, governors, and friends, may strengthen female entrepreneurs' network partnerships and quality assurances (Mazzarol et al., 2009). Thus, the negative impact of entrepreneurial experience on the creation of formal business plans is attenuated for female entrepreneurs. They are more likely than males to choose formal business planning, which may reduce the amount of risk and pressure they face.

In contrast, the gender difference in managerial experience is attenuated by proper corporate management. Under effective management systems, daily operations and maintenance are formally regulated. Female entrepreneurs' private time and space are guaranteed, helping them deal with risk, pressure, and work-life conflicts. Consequently, managerial experience does not influence male and female entrepreneurs differently than entrepreneurial experience, and gender moderation is not significant.

H5a Entrepreneurial experience has a significant differential impact on male and female entrepreneurs in choosing formal or informal business planning activities. Female entrepreneurs with entrepreneurial experience will be more likely to undertake formal business planning activities than male entrepreneurs with entrepreneurial experience.
H5b Managerial experience has no significant differential impact on male and female entrepreneurs in choosing formal or informal business planning activities.

Female entrepreneurs have a shortage of resources and social networks, which cause risk and uncertainty in the entrepreneurship process (Armua et al., 2020). With conservative strategies, they will undertake more entrepreneurship preparedness and gather as many resources as they can (Xie and Lv, 2018). Accordingly, female entrepreneurs with entrepreneurial experience prefer sophisticated planning because elaborate plans improve orientation and ease navigation in the early development stages of a new venture (Schulte, 2009). Furthermore, sophisticated plans are more persuasive and can facilitate capital acquisition accordingly. Female entrepreneurs with entrepreneurial experience are extremely sensitive to such opportunities and are more likely to engage in sophisticated planning.

**Figure 1:**
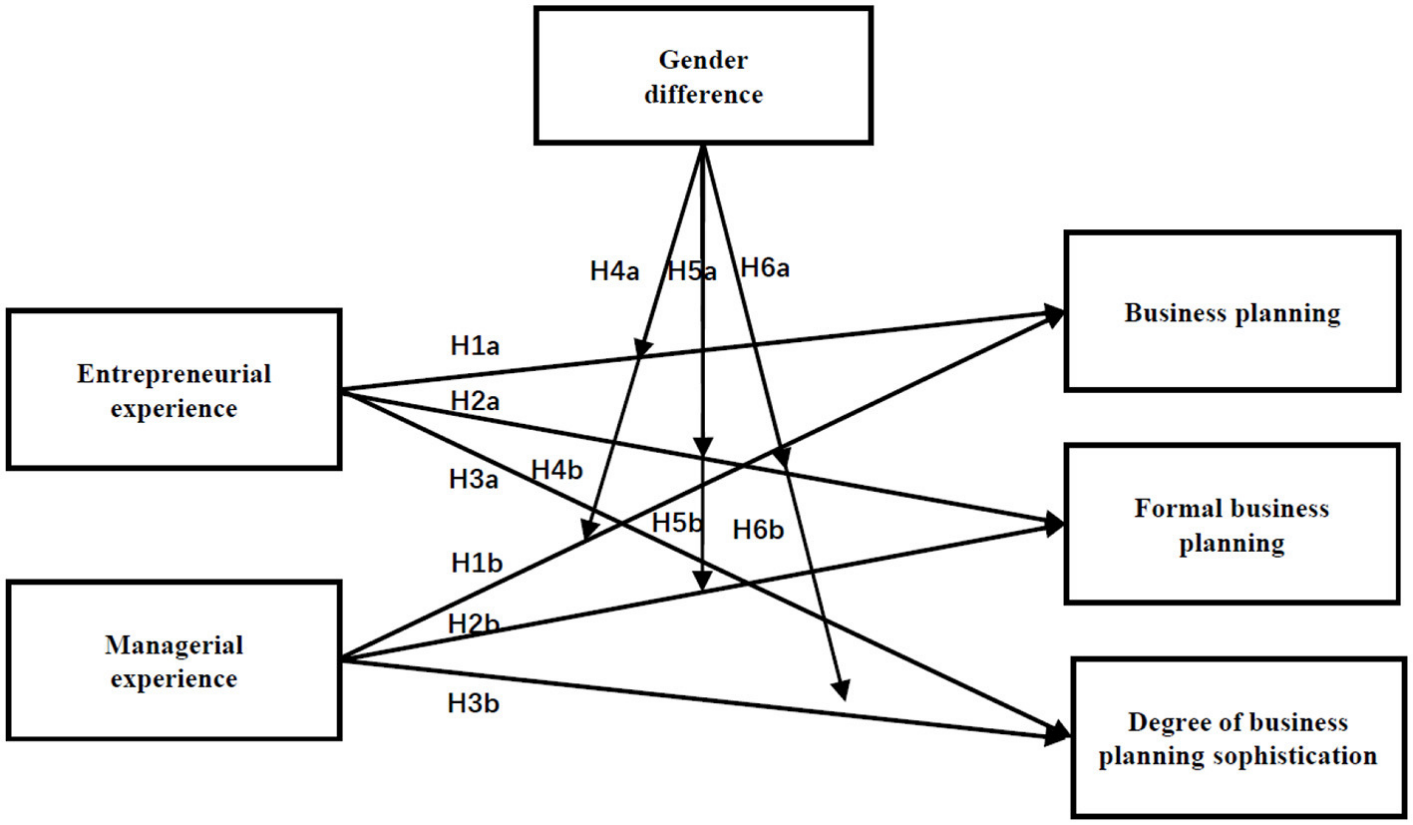
Conceptual model of the study.

Extensive planning activities and forecasts may lead to better decision quality but also induce higher costs (Brinckmann et al., 2010). However, for female entrepreneurs with managerial experience, their corporations pay the high costs of sophisticated planning. Accordingly, the positive relationship between female entrepreneurs and business planning sophistication is attenuated by managerial experience. The conceptual model described above is summarized in Figure 1.

H6a Entrepreneurial experience has a significant differential impact on male and female entrepreneurs in choosing their degree of business planning sophistication. Female entrepreneurs with entrepreneurial experience will be more likely to undertake sophisticated business planning activities than male entrepreneurs with entrepreneurial experience.
H6b Managerial experience has no significant differential impact on male and female entrepreneurs in choosing their degree of business planning sophistication.

## Method

### Literature Search

To identify and retrieve existing studies on business planning, we implemented a comprehensive and systematic search in a number of databases. First, we searched Google Scholar, EBSCO Host, the Web of Science, JSTOR, and ProQuest using the keywords “business planning,” “pre-startup planning,” or “business plan,” and we then added “experience,” “managerial experience” or “entrepreneurial experience.” Next, we conducted a search in the CNKI database, using our keywords to identify dissertations and papers in Chinese. Third, we manually searched the studies included in previous meta-analyses related to business planning (e.g., Brinckmann et al., 2010, 2019). In order to include unpublished studies such as dissertations, reports, book chapters, working papers and conference papers, we conducted searches in PsycINFO/Dissertation and ProQuest. The initial process yielded 24 references.

### Inclusion and Exclusion Criteria

This study followed an evidence-based research approach and applied meta-analysis. We included studies that measured one dimension of business planning, formal business plans; studies that measured the degree of planning sophistication; and studies that measured one type of experience that was defined in the present study. We excluded studies that reported one of the dimensions but did not focus on business plans in ventures (e.g., Gibbons and O'connor, 2005; Haber and Reichel, 2007; Mengel and Wouters, 2015). We further excluded studies measuring operational planning (e.g., Matthews and Scott, 1995), functional planning or financial planning (e.g., Mengel and Wouters, 2015). According to our inclusion criteria, we excluded review articles (e.g., Gonzalez, 2017) and studies with qualitative methodologies (e.g., Karlsson and Honig, 2009; Chwolka and Raith, 2012).

In addition, the criteria led to the exclusion of three studies that measured none of the experience variables (e.g., Bygrave et al., 2007; Englis et al., 2012; Zwerus, 2013). The studies we included had to report on the relationship between business planning and at least one type of experience or one of the two dimensions (formal business plan or degree of planning sophistication) and one type of experience. Finally, 32 articles are included in the meta-analysis and they are listed in the Appendix. We computed a composite score in the case of multiple dimensions that measured one construct. Specifically, when studies reported on the relationship between each of the two dimensions and one of the relevant correlates (experience), we used the comprehensive formula of Schmidt and Hunter (2015) to calculate a composite score across the dimension correlations to represent the overall business plan relationship.

### Coding Procedure

Two of the authors played the role of primary coder, and the third author acted as the secondary coder. At the very beginning of our coding stage, we developed a coding scheme according to Krippendorff (2012), and the two coders of this study independently coded the data following the developed coding scheme. For each paper, the authors coded the study's information, including the (1) sample size, (2) correlations, (3) reliability of each variable, and (4) sample characteristics, such as the percentage of males in the study and with which country the sample was associated.

## Measures

The measures for each experience dimension and business plan dimension (formal business plan and degree of planning sophistication) are inventoried in Table 1.

**Table 1:** Definitions of study measures.

**Experience**	**Business planning**		
**Managerial experience**	**Entrepreneurial experience**	**Formal business plan**	**Degree of planning sophistication**
Years management experience/ Years of Managerial Experience/ Managerial experience/ Management exp Managerial skills index/ CEO Experience/ Breadth of m. exp/ Management experience/	Prior entrepreneurial experience/ Previous start-up experience/ Start exp/ Start-up experience/ Entrepreneur owns another business/ First or subsequent venture (dummy)/ Start up exp/ Entrepreneurial experience/ Founding experience in years/ Prior Startup Experience/	W/without planning/ Business plan Plan/ Early planning/ Business Plan Presence/ Doing business planning or not/ No. of planning competitions/ No. of business plans/ Has a business plan been prepared for? /	Formal business planning/ Changed Business Plan/ Business plan completeness/ Formal Planners/ The quality of business planning/ Planning sophistication/ Planning opportunity/ Total BP pages/ Informal Business planning/ Planning marketing mix/ Systematic-planner/ Formalized Business Plan/ Formal presentations operational planning/

### Business Plan

Based on a prior study on business planning (e.g., Liao and Gartner, 2007), we contained a mass of planning-related measures. We categorized business plan measures into “formal business plan” and “degree of planning sophistication.” For overall business plans, relationships were either coded directly from primary studies or combined from dimension-level relationships using a composite formula (Schmidt and Hunter, 2015).

### Experience

Entrepreneur experience refers to an entrepreneur's various skills, knowledge, and rational or perceptual concepts. Scholars like Politis (2005) and Ucbasaran et al. (2009) have divided entrepreneur experience into entrepreneurial experience, managerial experience, and functional experience. Unger et al. (2011) divided entrepreneur experience into entrepreneurial experience, managerial experience, and work experience. In addition, work experience has been most frequently used to assess no-task-related human capital. Thus, in order to distinguish the impact of different experiences on the dimensions of business planning activity, we classified the experience measure into two categories: managerial experience and entrepreneurial experience. Following previous research, we contained relationships when they were represented in at least three independent samples.

### Moderator

Gender was dummy coded such that higher values were indicative of males (i.e., 0 = female, 1 = male). To evaluate how gender moderated the experience-business plan relationship, we used a gender ratio (i.e., the percentage of each sample that was male).

### General Meta-Analytic Procedures

Based on the literature search, in the first step, we coded the studies according to the a priori developed coding protocol and checked the coded data. Disagreements were discussed until we reached a consensus. In the second step, we used the Comprehensive Meta-Analysis software (CMA 3.0) to calculate the effect size using the random-effects model suggested by Hedges and Olkin (1985) because most of the variables in our study were observed variables. For each relationship between job crafting and outcomes, we reported the independent effect size (k), sample size (N), weighted mean correlation (r), and 95% confidence interval for the mean effect. We also reported the Q statistic to quantify heterogeneity and the standard deviation of true effect sizes (T). The result was considered statistically significant if its confidence interval excluded zero. In the third step, we added the moderator variable and analyzed the influence on the experience-business plan relationship by using meta-regressions.

## Results

Meta-analysis offered an empirical opportunity to explore experience and business planning relationships. The results of our meta-analysis are presented in Table 2. We also studied the relationship between formal business planning activity and the degree of business planning sophistication and entrepreneurial experience and managerial experience.

**Table 2:** Meta-analysis of relationships between business plan and experience.

**Variables**	**K**	**N**	$\bar{r}$	**95% CI**		**Q**	**P**
				**LL**	**UL**		
Business experience—Business planning	32	11064	0.069	0.03	0.107	117.18	0.000
H1a: Entrepreneurial experience—Business planning	21	7880	0.05	0.019	0.08	32.112	0.042
H1b: Managerial experience—Business planning	11	3291	0.132	0.03	0.23	76.726	0.000
H2a: Entrepreneurial experience—Formal business planning	10	3261	−0.005	−0.046	0.035	11.65	0.234
H2b: Managerial experience—Formal business planning	8	3191	0.07	0.005	0.135	23.538	0.001
H3a: Entrepreneurial experience—Degree of business planning sophistication	13	5791	0.075	0.048	0.101	12.504	0.406
H3b: Managerial experience—Degree of business planning sophistication	8	2428	0.137	0.028	0.244	48.469	0.000

*K, cumulative number of studies; N, cumulative sample size; $\bar{r}$, point estimate (random); 95% CI, 95% confidence interval for $\bar{r}$; LL, lower level of the 95% CI; UL, upper level of the 95% CI*.

To test hypothesis 1a, the relationship between entrepreneurial experience and business planning activity was examined. Entrepreneurial experience correlated positively with business planning activity ($\bar{r}\;=$ 0.05). Therefore, the results supported hypothesis 1a. Similarly, managerial experience ($\bar{r}$ = 0.132) correlated positively with business planning activity. Therefore, the results supported hypothesis 1b.

To test hypothesis 2a and hypothesis 2b, we examined the relationships between formal business planning activity and managerial and entrepreneurial experience. The results showed that managerial experience correlated positively with formal business plans ($\bar{r}$ = 0.07), whereas entrepreneurial experience ($\bar{r}$ = −0.005) had no significant relationship with formal business plans, as shown in Table 2. Thus, hypothesis 2b was supported, while hypothesis 2a was rejected.

Based on hypotheses 3a and 3b, the relationships between entrepreneurial experience and managerial experience and the degree of business planning sophistication were examined. Table 2 shows that entrepreneurial experience ($\bar{r}$ = 0.075) correlated positively with the degree of business planning sophistication. Managerial experience also correlated positively with the degree of business planning sophistication ($\bar{r}$ = 0.137). Hence, hypotheses 3a and 3b were also supported.

To test hypotheses 4a and 4b, we used the proportion of men that each study reported and analyzed the moderating effect of the sample gender on the relationships and subrelationships between business planning activity and experience through meta-regression models. We present the results of the meta-regression analyses in Table 3. Across each of the models considered, the slope for gender (B = −0.319) for the relationship between business planning activity and entrepreneurial experience was negative, suggesting that the business planning activity-entrepreneurial experience relationship was stronger among the samples of females. Meanwhile, we found that gender (B = 0.446) had no significant moderating effect on the relationship between business planning activity and managerial experience. Thus, hypothesis 4a and hypothesis 4b were supported.

**Table 3:** Moderating effect of gender variables of business plan-experience relationships.

**Moderator**	**Relationships**	**B**	**SE**	**95% CI**		**Z-value**	***P*-value**
				**LL**	**UL**		
Gender	Business planning—experience	−0.049	0.120	−0.279	0.182	−0.413	0.679
	H4a: Entrepreneurial experience—Business planning	−0.319	0.128	−0.571	−0.067	−2.481	0.013
	H4b : Managerial experience—Business planning	0.446	0.426	−0.389	1.280	1.047	0.295
	H5a: Entrepreneurial experience—Formal business planning	−0.531	0.265	−1.020	−0.011	−2.001	0.045
	H5b: Managerial experience—Formal business planning	0.009	0.388	−0.750	0.769	0.024	0.981
	H6a: Entrepreneurial experience— Degree of business planning sophistication	−0.205	0.152	−0.502	0.092	−1.351	0.177
	H6b: Managerial experience—Degree of business planning sophistication	0.261	0.406	−0.535	1.057	0.643	0.520

*B, regression coefficient for moderator; SE, standard error; 95% CI, 95% confidence interval; LL, lower level of the 95% CI; UL, upper level of the 95% CI*.

To test hypotheses 5a and 5b, we analyzed the moderating effect of gender on the relationships between formal business planning activity and entrepreneurial experience and managerial experience. The slope for gender (B = −0.531) for the relationship between formal business planning activity and entrepreneurial experience was negative, implying that the relationship was weaker among the samples of males. Thus, hypothesis 5a was supported. The moderating effect of gender (B=0.009) between managerial experience and the degree of business planning sophistication was insignificant. Thus, hypothesis 5b was supported.

To test hypotheses 6a and 6b, we analyzed the moderating effect of gender on the relationships between the degree of business planning sophistication and entrepreneurial experience and managerial experience. The slope for gender (B = −0.205) for the relationship between the degree of business planning sophistication and entrepreneurial experience was negative but non-significant. This implied that gender had no significant effect on the relationship. Thus, hypothesis 6a was rejected. The moderating effect of gender (B = 0.261) on the relationship between managerial experience and the degree of business planning sophistication was insignificant. This implied that managerial experience has no significant differential impact on male and female entrepreneurs in choosing their degree of business planning sophistication. Thus, hypothesis 6b was supported.

## Discussion

This article addresses a fundamental issue, that is, why entrepreneurs behave differently in the business planning process. To date, entrepreneurial preparedness research has lacked a theory to explain entrepreneurs' disposition to business planning behaviors. In this study, we drew insight from institutional theory and effectuation theory and meta-analytically analyzed the relationships between different types of entrepreneur experiences and business planning. In addition, we tested the gender difference in business planning and examined the moderating role of gender on these relationships.

We found that entrepreneurs with business experience are more likely to undertake business planning (formal/informal). This result offers the novel insight that business plans are not symbolic documents (Karlsson and Honig, 2009) but adopted as useful tools by experienced entrepreneurs to deal with high uncertainty and risk. We empirically verified that business experience, including entrepreneurial experience and managerial experience, increases entrepreneurs' willingness to engage in business planning. This result, to some extent, is contrary to some scholars' arguments that prior experience will decrease entrepreneurs' motivation to undertake business planning as they possess more knowledge and skills (Dencker et al., 2009; Rotger et al., 2012; Borges et al., 2013). We propose that, through prior business operation, entrepreneurs acknowledge that good preparedness and systematic business planning are desirable for venture creation. On the one hand, entrepreneurship processes cannot be replicated, and prior knowledge is difficult to transfer to the subsequent venture creation processes. On the other hand, prior business experience may teach entrepreneurs that entrepreneurship involves a high level of uncertainty and extraordinary risk and that entrepreneurial preparedness is an indispensable activity.

### Theoretical Implications

Through our theorizing and analysis, we make three primary contributions to the extant entrepreneurial planning literature. First, rather than assess the value of business planning for venture performance (Delmar and Shane, 2003; Gruber, 2007; Burke et al., 2010), we extend this stream of research by addressing why business planning should be undertaken and to what extent. We separate business planning into different forms rather than lump them into a dummy variable (plan or no plan). As most previous studies have tried to account for why entrepreneurs plan or not but have neglected the idea that many entrepreneurs plan in their heads or only generate basic plans, many of the conclusions drawn in those studies are ambiguous and conflict with each other. The present study not only discusses whether entrepreneurs plan or not but also gives attention to whether they choose formal or informal business planning and their degree of business planning sophistication. Although “plan in head” involves no physical behavior and is very similar to not planning, these two activities are totally different. Contradicting the institutional perspective that business planning is a symbolic exercise and a non-economically rational activity (Karlsson and Honig, 2009), entrepreneurs with business experience acknowledge the value of business planning and are more likely to undertake business planning (formal or informal). Thus, the motivation to make a business plan is not attenuated by business experience (Borges et al., 2013) but reinforced.

Second, the extant literature always highlights the value of business planning but ignores the different costs of formal and informal business plans. This study provides a quantitative synthesis of empirical studies and explains why many entrepreneurs prefer to “plan in head” from the effectuation perspective. Our study shows that entrepreneurs with entrepreneurial experience prefer informal business plans, whereas entrepreneurs with management experience prefer formal written business plans. Although we failed to find support for hypothesis H2a at P < 0.05, the correlated r for entrepreneurial experience and formal business planning was negative, showing a negative relationship between entrepreneurial experience and formal business planning. Those entrepreneurs not only recognize the value of business planning but also calculate the planning cost. If entrepreneurs determine that formal written business plans cost too much, they will choose “simple,” “cheap,” and “convenient” approaches (Sarasvathy, 2001; Dew et al., 2009). In contrast, the institutional context forces managers to behave in a more systematic and premeditated manner with corporate budgets. More experienced managers are expected to have a more formal approach to business planning in their business behavior (Richbell et al., 2006). Accordingly, they believe that the benefits of formal business planning outweigh the related costs.

Finally, by drawing on the gender moderation effect, this paper provides a bridge between the business planning literature and gender differences. Although many existing studies have discussed gender inequality and business planning behaviors, there are few empirical studies. To fill this gap, we investigated the moderating effect of gender, primarily focusing on whether entrepreneurial experience and managerial experience influence male and female entrepreneurs equally or not. Surprisingly, we found that entrepreneurial experience had a significantly different effect on male and female entrepreneurs, but this gender difference did not exist in the context of managerial experience. We found that female entrepreneurs with entrepreneurial experience, who avoid risk and fear uncertainty, are more likely to engage in business planning behaviors and formal business plans.

### Practical Implication

Our findings provide several practical implications. First, we should educate nascent entrepreneurs that business planning will bring benefits. However, at the same time, we should tell them that business planning incurs cost as well. A sophisticated written business plans may not be suitable for everyone. Entrepreneurs should undertake business planning in correspondence to their specific situations. Second, entrepreneurial experience and managerial experience may make entrepreneurs have bias toward specific business planning behavior. Entrepreneurs with managerial experience may overestimate the business planning while entrepreneurs with entrepreneurial experience may underappreciate business planning. Accordingly, it may be helpful to remind entrepreneurs about cognitive biases in offsetting their biases. Third, based on the gender interaction test, we find that female entrepreneurs are affected by cognitive biases more seriously. Female entrepreneurs with entrepreneurial experience are more likely to undertake business-planning behaviors and create formal business plans. Hence, educators should be more cautious when educating female entrepreneurs and developing a more contextual understanding of applying different approaches rather than advocating only planning (Brinckmann et al., 2019).

### Limitations and Future Research

Complex factors influence business planning. Our paper only investigated the influence of experience and gender and could be criticized for over-reduction. More environmental factors and individual characteristics should be investigated in the future.

Another limitation of this study is that meta-analysis relies on the specifications of the underlying studies. Although meta-analysis is an important method for making empirical estimates, the original studies may have influenced our analysis. It should be noted that some interpretations in the analysis process are perceptually measured as scholars depict and express personalization. In addition, we focused on entrepreneurs who engaged in business planning, but the explanation of why entrepreneurs do not plan was not addressed.

In addition, our keywords for searching process are not completely exhaustive. There are many synonyms for business planning. Although we endeavor to identify every potential research, we may miss some related studies and not all targets are included in our sample.

With respect to the antecedents of business planning, future research could examine other contextual factors. Additionally, factors such as individual personality, organizational culture, and academic education may also be important factors that determine business planning behaviors. Furthermore, different degrees of planning formalization may impact new ventures differently while evolving over time. However, we still do not have clarity about the mechanism.

## Conclusion

Drawing upon the meta-analysis, this study examined how entrepreneurial experience and managerial experience affect entrepreneurs' planning behaviors. Specifically, we found that entrepreneurial experience and managerial experience increase entrepreneurs' willingness to engage in business planning. In addition, entrepreneurs with entrepreneurial experience prefer informal business plans, whereas entrepreneurs with managerial experience prefer formal written business plans. Both entrepreneurial experience and managerial experience positively influence the degree of planning sophistication. For the moderating effects of gender, female entrepreneurs, who avoid risk and fear uncertainty, are more likely to engage in business planning.

The value of business planning for the performance of firms is broadly examined in academic research. However, empirical findings have been fragmented and contradictory (Brinckmann et al., 2010). Although we cannot solve the theoretical debate, this study provides evidence that experienced entrepreneurs acknowledge the value of business planning.

## Data Availability Statement

The original contributions presented in the study are included in the article, further inquiries can be directed to the corresponding authors.

## Ethics Statement

Ethical review and approval was not required for the study as this is a meta analysis in which no human participants have been involved in this study.

## Author Contributions

JM designed the overall research, collected data, and analyzed data. SC conducted literature review and interpreted the data. YW designed the entire framework, supervised the study, and made substantive changes. MS helped the first author to analyze data. All authors contributed equally to this manuscript, and reviewed and approved this manuscript for publication.

## Conflict of Interest

The authors declare that the research was conducted in the absence of any commercial or financial relationships that could be construed as a potential conflict of interest.

## Publisher's Note

All claims expressed in this article are solely those of the authors and do not necessarily represent those of their affiliated organizations, or those of the publisher, the editors and the reviewers. Any product that may be evaluated in this article, or claim that may be made by its manufacturer, is not guaranteed or endorsed by the publisher.
