# Supplementary material for: The Role of Experience and Gender in Founders' Business Planning Activities: A Meta Analysis
Source: Front Psychol. 2021 Aug 12;12:689632. doi: 10.3389/fpsyg.2021.689632 (PMC8387563; doi:10.3389/fpsyg.2021.689632)
Supplement: Supplementary file 1 [file Data_Sheet_1.docx]

Appendix Articles included in meta-analysis

*Burke, A. E., Fraser, S., & Greene, F. J. (2010). The Multiple Effects of Business Planning on New Venture Performance. *J. Manage. Stud. 47*(3), 391-415. <https://doi.org/10.1111/j.1467-6486.2009.00857.x>

*Chaganti, R. S., Joy A. (1994). A study of the impact of owner's mode of entry on venture performance and management patterns. *J. Bus. Ventur. 9*(3), 243-260. <http://dx.doi.org/10.1016/0883-9026(94)90032-9>

*Chell, E. (2001). Entrepreneurship: globalization, innovation and development. *Int. J. Entrep. Behav. Res. 7*(5), 205-206.

*Contín-Pilart, I., Larraza-Kintana, M., & Martín-Sánchez, V. (2014). The Impact of Entrepreneurss Planning Profiles on Firm Growth: An Empirical Analysis. *SSRN.Electron. J.*  <http://dx.doi.org/10.2139/ssrn.2479916>

*Delmar, F., & Shane, S. (2003). Does business planning facilitate the development of new ventures? *Strateg. Manage. J. 24*(12), 1165-1185. https://doi.org/10.1002/smj. 349

*Delmar, F., & Shane, S. (2004). Legitimating first: organizing activities and the survival of new ventures. *J. Bus. Ventur. 19*(6), 385-410. <https://doi.org/10.1016/S0883-9026(03)00037-5>

*Dencker, J. C., Gruber, M., & Shah, S. K. (2009). Pre-Entry Knowledge, Learning, and the Survival of New Firms. *Organ Sci. 20*(3), 516-537. https://doi.org/10.1287/ orsc.1080.0387

*Dimov, D. (2010). Nascent Entrepreneurs and Venture Emergence: Opportunity Confidence, Human Capital, and Early Planning. *J. Manage. Stud. 47*(6), 1123-1153. <https://doi.org/10.1111/j.1467-6486.2009.00874.x>

*Duchesneau, D. A., & Gartner, W. B. (1990). A profile of new venture success and failure in an emerging industry. *J. Bus. Ventur. 5*(5), 297-312. https://doi.org/ 10.1016/0883-9026(90)90007-G

*Gavin, & Cassar. (2010). Are individuals entering self-employment overly optimistic? an empirical test of plans and projections on nascent entrepreneur expectations. *Strateg. Manage. J. 31*(8), 822-840. <https://doi.org/10.1002/smj.833>

*Gelderen, M. V., & Thurik, F. R. (2000). Strategies, Uncertainty and Performance of Small Business Startups. *Small Bus. Econ. 15*(3), 165-181. https://doi.org/10.1023/A: 1008113613597

*Gibbons, P. T., & O'Connor, T. (2005). Influences on Strategic Planning Processes among Irish SMEs. *J. Small Bus. Manag. 43*(2), 170-186. https://doi.org/10.1111/ j.1540-627x.2005.00132.x

*Gielnik, M. M., Barabas, S., Frese, M., Namatovu-Dawa, R., Scholz, F. A., Metzger, J. R., & Walter, T. (2014). A temporal analysis of how entrepreneurial goal intentions, positive fantasies, and action planning affect starting a new venture and when the effects wear off. *J. Bus. Ventur. 29*(6), 755-772. <https://doi.org/10.1016/j.jbusvent>. 2013.09. 002

*Greene, F. J., & Hopp, C. (2017). Are formal planners more likely to achieve new venture viability?: A counterfactual model and analysis. *Strateg. Entrep. J. 11*(1), 36-60. <https://doi.org/10.1002/sej.1245>

*Haber, S., & Reichel, A. (2007). The cumulative nature of the entrepreneurial process: The contribution of human capital, planning and environment resources to small venture performance. *J. Bus. Ventur. 22*(1), 119-145. https://doi.org/10.1016/j. jbusvent.2005.09.005

*Hiatt, S. R., & Sine, W. D. (2014). Clear and present danger: Planning and new venture survival amid political and civil violence. *Strateg. Manage. J. 35*(5), 773-785. <https://doi.org/10.1002/smj.2113>

*Honig, B., & Karlsson, T. (2013). An Institutional Perspective on Business Planning Activities for Nascent Entrepreneurs in Sweden and the US. *Adm. Sci. 3*(4), 266-289. <https://doi.org/10.3390/admsci3040266>

*Hopp, C. (2015). Does the presence of a formal business plan increase formal financial support? Empirical evidence from the PSED II on the signalling and mimetic nature of formal business planning. *Appl. Econ. Lett. 22*(9), 673-678. https://doi.org/10.1080/13504851. 2014.967377

*Karlsson, T., & Honig, B. (2004). Institutional forces and the written business plan. *J. Manag. 30*(1), 29-48. <https://doi.org/10.1016/j.jm.2002.11.002>

*Lange, J. E., Mollov, A., Pearlmutter, M., Bygrave, S. S., & D., W. (2007). Pre-start-up formal business plans and post-start-up performance: A study of 116 new ventures. *Ventur. Cap. 9*(4), 237-256. <https://doi.org/10.1080/13691060701414840>

*Liao, J., & Gartner, W. B. (2006). The Effects of Pre-venture Plan Timing and Perceived Environmental Uncertainty on the Persistence of Emerging Firms. *Small Bus. Econ. 27*(1), 23-40. 10.1007/s11187-006-0020-0

*Liao, J., & Gartner, W. B. (2007). The Influence of Pre-Venture Planning on New Venture Creation. *J. Small Bus.Strateg. 18*(2), 1-22.

*Liao, J., & Gartner, W. B. (2009). *Are Planners Doers Pre-venture Planning and the Start-up Behaviors of Entrepreneurs*. SBA Office of Advocacy.

*Lussier, R. N. (1995). A nonfinancial business success versus failure prediction model for young firms. *J. Small Bus. Manag. 33*(1), 8-20.

*Lussier, R. N. (1996). A Business Success versus Failure Prediction Model for Service Industries. *J. Bus.Entrep. 8*(2), 23-37.

*Matthews, C. H., Schenkel, M. T., Ford, M. W., & Human, S. E. (2009). Comparing Nascent Entrepreneurs and Intrapreneurs and Expectations of Firm Growth. *J. Small Bus.Strateg. 20*(2), 53-80.

*McCann, B. T., & Vroom, G. (2015). Opportunity evaluation and changing beliefs during the nascent entrepreneurial process. *Int. Small Bus. J. 33*(6), 612-637. <https://doi.org/10.1177/0266242614544198>

*Mengel., & Wouters. (2015). Financial planning and control in very small start-up companies: antecedents and effects on company performance. *Int. J. Entrep.Small Bus. 26*(2), 191-216. <https://doi.org/10.1504/IJESB.2015.071824>

*Park, B. I. (2010). What matters to managerial knowledge acquisition in international joint ventures? High knowledge acquirers versus low knowledge acquirers. *Asia Pacific J. Manag. 27*(1), 55-79. <https://doi.org/10.1007/s10490-008-9111-6>

*Shane, S., & Delmar, F. (2004). Planning for the market: business planning before marketing and the continuation of organizing efforts. *J. Bus. Ventur. 19*(6), 767-785. <https://doi.org/10.1016/j.jbusvent.2003.11.001>

*Thiele, P., & Fellnhofer, K. (2015). The impact of pre-startup planning on the strength of planning assumptions and the mode of processing. *Int. J. Entrep.Ventur. 7*(2), 173-193. <https://doi.org/10.1504/IJEV.2015.068601>

*Zhang, Y., Yang, J., Tang, J., Au, K., & Xue, H. (2013). Prior Experience and Social Class as Moderators of the Planning-Performance Relationship in China's Emerging Economy. *Strateg. Entrep. J. 7*(3), 214-229. <https://doi.org/10.1002/sej.1155>
